# BTK Has Potential to Be a Prognostic Factor for Lung Adenocarcinoma and an Indicator for Tumor Microenvironment Remodeling: A Study Based on TCGA Data Mining

**DOI:** 10.3389/fonc.2020.00424

**Published:** 2020-04-15

**Authors:** Ke-Wei Bi, Xu-Ge Wei, Xiao-Xue Qin, Bo Li

**Affiliations:** Key Laboratory of Cell Biology, Department of Developmental Cell Biology, Ministry of Public Health and Key Laboratory of Medical Cell Biology, Ministry of Education, China Medical University, Shenyang, China

**Keywords:** BTK, tumor microenvironment, ESTIMATE, CIBERSORT, tumor-infiltrating immune cells, lung adenocarcinoma

## Abstract

Tumor microenvironment (TME) plays a crucial role in the initiation and progression of lung adenocarcinoma (LUAD); however, there is still a challenge in understanding the dynamic modulation of the immune and stromal components in TME. In the presented study, we applied CIBERSORT and ESTIMATE computational methods to calculate the proportion of tumor-infiltrating immune cell (TIC) and the amount of immune and stromal components in 551 LUAD cases from The Cancer Genome Atlas (TCGA) database. The differentially expressed genes (DEGs) were analyzed by COX regression analysis and protein–protein interaction (PPI) network construction. Then, Bruton tyrosine kinase (BTK) was determined as a predictive factor by the intersection analysis of univariate COX and PPI. Further analysis revealed that BTK expression was negatively correlated with the clinical pathologic characteristics (clinical stage, distant metastasis) and positively correlated with the survival of LUAD patients. Gene Set Enrichment Analysis (GSEA) showed that the genes in the high-expression BTK group were mainly enriched in immune-related activities. In the low-expression BTK group, the genes were enriched in metabolic pathways. CIBERSORT analysis for the proportion of TICs revealed that B-cell memory and CD8+ T cells were positively correlated with BTK expression, suggesting that BTK might be responsible for the preservation of immune-dominant status for TME. Thus, the levels of BTK might be useful for outlining the prognosis of LUAD patients and especially be a clue that the status of TME transition from immune-dominant to metabolic activity, which offered an extra insight for therapeutics of LUAD.

## Introduction

Lung cancer is the main cause of cancer-related death worldwide by reason of high recurrence rate, late detection, and poor prognosis. As a member of non-small cell lung cancer (NSCLC), LUAD accounts for ~40% of all lung cancer cases. The current treatment for lung cancer, including surgical resection, chemotherapy, and radiation, had limitation on the improvement of patient's survival (1, 2). Accordingly, it is urgently needed to explore the carcinogenesis and therapeutics of lung cancer.

Increasing evidence demonstrated the importance of the tumor microenvironment (TME) in the tumor development. Collaborative interactions between cancer cells and their supporting cells contributed to the malignant phenotypes of cancer, such as immortal proliferation, resisting apoptosis, and evading immune surveillance. Therefore, the TME significantly influences therapeutic response and clinical outcome in cancer patients (3, 4). Structural components of the TME are mainly resident stromal cells and recruited immune cells. While there was compelling evidence for the role of stromal cell contributing to tumor angiogenesis and extracellular matrix remodeling, but perhaps it is still not fully understood (5). Meanwhile, a few studies paid close attention to the impact of the immune cells in TME on tumor growth and progression. A growing body of studies showed that the tumor-infiltrating immune cell (TIC) in TME served as a promising indicator for the therapeutic effects (6). The tumor-infiltrating lymphocyte (TIL) was significantly correlated with the 5-year survival of NSCLC, and low lymphocyte abundance in cancer was identified as a poor prognostic indicator in early-stage NSCLC (7, 8). This relevance brought about the improvement of immune-based therapeutics, resulting in the application of immune checkpoint inhibitors for NSCLC patients (9, 10). A recent study elucidated the role of lung cancer lineage specifiers SOX2 and NKX2-1 in tumor cell fate and neutrophil recruitment, suggesting that the determination of tumor immune microenvironment might impact the nature of the tumor (11). A gene-expression profiling analysis showed that immune activation and immune escape in TME occur before lung cancer invasion (12). These results suggested that the adaptive immune response within TME might be of much importance at the earliest stage of lung cancer. Therefore, there is a challenge in performing precise genetic analysis that could appropriately indicate the dynamic modulation of the immune and stromal components in TME.

Transcriptome-sequencing patterns followed by functional genomics analysis have shed light on the roles of different types of cells during TME modulation. In the presented article, we applied ESTIMATE and CIBERSORT computational methods to calculate the TIC proportion and the ratio of immune and stromal components of LUAD samples from The Cancer Genome Atlas (TCGA) database and identified a predictive biomarker, Bruton tyrosine kinase (BTK). BTK was a non-receptor tyrosine kinase of the Tec family, locating in the downstream of signal transduction of B-cell antigen receptor (BCR). Upon phosphorylated, BTK triggered several signaling pathways, which resulted in the survival of leukemic cells in many B-cell malignancies (13, 14). Recently, some preclinical data had demonstrated that BTK was overexpressed in some solid tumors and their periphery cells in TME such as dendritic cells, macrophages, myeloid derived suppressor cells, and endothelial cells (15, 16), suggesting that BTK might play a role in TME. Here we embarked from differentially expressed genes (DEGs) generated by comparison between immune components and stromal components in LUAD samples and revealed that the BTK might be a potential indicator for the alteration of TME status in LUAD.

## Results

### Analysis Process of This Study

**Figure 1:**
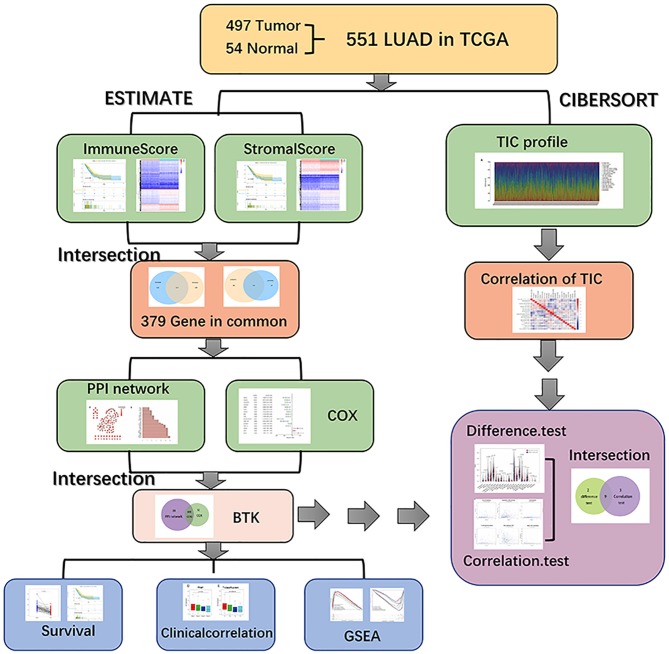
Analysis workflow of this study.

The analysis process of our study is shown in in Figure 1. To estimate the proportion of TICs and the amount of immune and stromal component in LUAD samples, transcriptome RNA-seq data of 551 cases were downloaded from TCGA database followed by calculating with CIBERSORT and ESTIMATE algorithms. DEGs shared by ImmuneScore and StromalScore were used to constructed protein–protein interaction (PPI) network and univariate COX regression analysis, and then intersection analysis was performed using the core nodes in PPI network and the top significant factors obtained from the analysis of univariate COX regression. BTK and CCR2 were obtained, and we focused on BTK for the subsequent series of analysis, including survival and clinicopathological characteristics correlation analysis, COX regression, Gene Set Enrichment Analysis (GSEA), and correlation with TICs.

### Scores Were Correlated With the Survival of LUAD Patients

**Figure 2:**
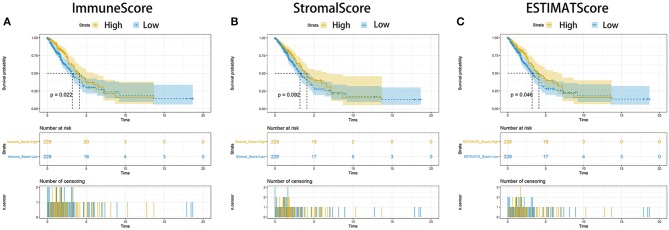
Correlation of scores with the survival of patients with LUAD. **(A)** Kaplan–Meier survival analysis for LUAD patients grouped into high or low score in ImmuneScore determined by the comparison with the median. *p* = 0.022 by log-rank test. **(B)** Kaplan–Meier survival curve for StromalScore with *p* = 0.092 by log-rank test. **(C)** Survival analysis with Kaplan–Meier method for LUAD patients grouped by ESTIMATEScore (*p* = 0.046 by log-rank test).

To establish the correlation of the estimated proportion of immune and stromal with the survival rate, Kaplan–Meier survival analysis was used for ImmuneScore, StromalScore, and ESTIMATEScore, respectively. The higher score estimated in ImmuneScore or StromalScore were represented for the larger amount of the immune or stromal components in TME. ESTIMATEScore was the sum of ImmuneScore and StromalScore denoting the comprehensive proportion of both components in TME. As shown in Figure 2A, the proportion of immune components had positive correlation with the overall survival rate. Despite StromalScore had no significant correlation with the overall survival rate (Figure 2B), ESTIMATEScore still showed positive correlation with the survival rate (Figure 2C). These results implied that the immune components in TME were more suitable for indicating the prognosis of LUAD patients.

### Scores Were Associated With the Clinic–Pathological Staging of LUAD Patients

**Figure 3:**
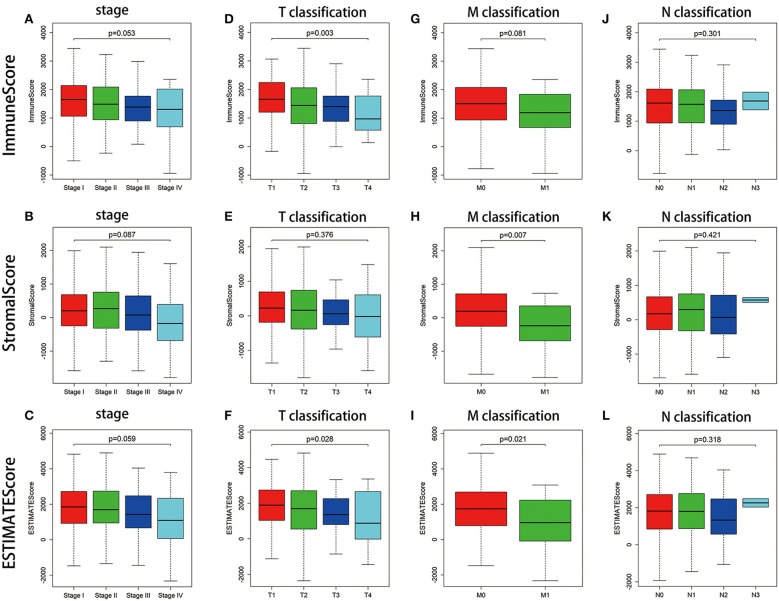
Correlation of ImmuneScore and StromalScore with clinicopathological staging characteristics. **(A–C)** Distribution of ImmuneScore, StromalScore, and ESTIMATEScore in stage. The *p* = 0.053, 0.087, and 0.059, respectively, by Kruskal–Wallis rank sum test. **(D–F)** Distribution of three kinds of scores in T classification (*p* = 0.003, 0.376, 0.028 for ImmuneScore, StromalScore, and ESTIMATEScore, respectively, by Kruskal–Wallis rank sum test). **(G–I)** Distribution of scores in M classification (*p* = 0.081, 0.007, 0.021 for ImmuneScore, StromalScore, and ESTIMATEScore separately by Wilcoxon rank sum test). **(J–L)** Distribution of scores in N classification. Similar to the preceding, *p* = 0.301, 0.421, 0.318, respectively, with Kruskal–Wallis rank sum test.

For determining the relationship between the proportion of immune and stromal components with the clinicopathological characteristics, we analyzed the corresponding clinical information of LUAD cases from TCGA database. As shown in Figure 3, ImmuneScore showed the negative correlation with T classification of TMN stages (Figure 3D, *p* = 0.003); StromalScore was only negatively correlated to M classification of TMN stages (Figure 3H, *p* = 0.007), and ESTIMATEScore significantly declined accompany with the advance of TMN stages (Figure 3F, *p* = 0.028 and Figure 3I, *p* = 0.021). These results suggested that the ratio of immune and stromal components was associated with the progress of LUAD, such as invasion and metastasis.

### DEGs Shared by ImmuneScore and StromalScore Were Predominantly Presented as the Enrichment of Immune-Related Genes

**Figure 4:**
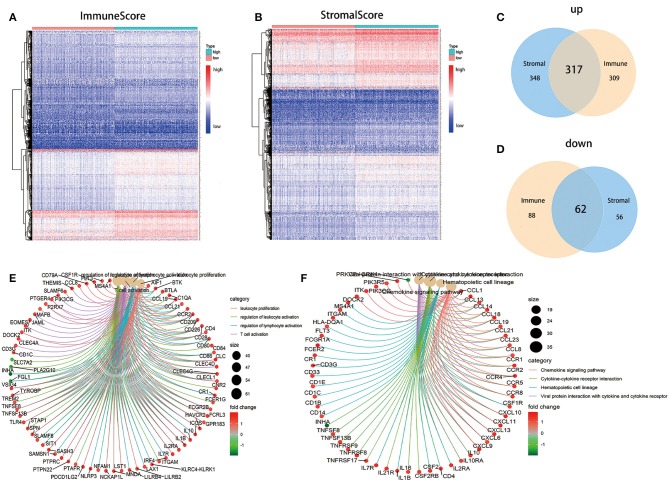
Heatmaps, Venn plots, and enrichment analysis of GO and KEGG for DEGs. **(A)** Heatmap for DEGs generated by comparison of the high score group vs. the low score group in ImmuneScore. Row name of heatmap is the gene name, and column name is the ID of samples which not shown in plot. Differentially expressed genes were determined by Wilcoxon rank sum test with *q* = 0.05 and fold-change >1 after log_2_ transformation as the significance threshold. **(B)** Heatmap for DEGs in StromalScore, similar with **(A)**. **(C,D)** Venn plots showing common up-regulated and down-regulated DEGs shared by ImmuneScore and StromalScore, and *q* < 0.05 and fold-change >1 after log_2_ transformation as the DEGs significance filtering threshold. **(E,F)** GO and KEGG enrichment analysis for 379 DEGs, terms with *p* and *q* < 0.05 were believed to be enriched significantly.

To ascertain the exact alterations of gene profile in TME regarding immune and stromal components, the comparison analysis between high- and low-score samples were carried out. Compared to the median, the total 776 DEGs were obtained from ImmuneScore (samples with high score vs. low score) Among them, 626 genes were up-regulated, and 150 genes were down-regulated (Figures 4A,C,D). Similarly, 783 DEGs were obtained from StromalScore, consisting of 665 up-regulated genes and 118 down-regulated genes (Figures 4B–D). The intersection analysis displayed by Venn plot showed a total of 317 up-regulated genes sharing by high score both in ImmuneScore and StromalScore and 62 down-regulated genes sharing by low score as well. These DEGs (total 379 genes) were possibly determinate factors for the status of TME. Results from gene ontology (GO) enrichment analysis indicated that the DEGs almost mapped to the immune-related GO terms, such as leukocyte proliferation and T-cell activation (Figure 4E). The Kyoto Encyclopedia of Genes and Genomes (KEGG) enrichment analysis also displayed the enrichment of chemokine signaling pathway, cytokine–cytokine receptor interaction, and hematopoietic cell lineage (Figure 4F). Thus, the overall functions of DEGs seemed to map on immune-related activities, which implied that the involvement of immune factors was a predominant feature of TME in LUAD.

### Intersection Analysis of PPI Network and Univariate COX Regression

**Figure 5:**
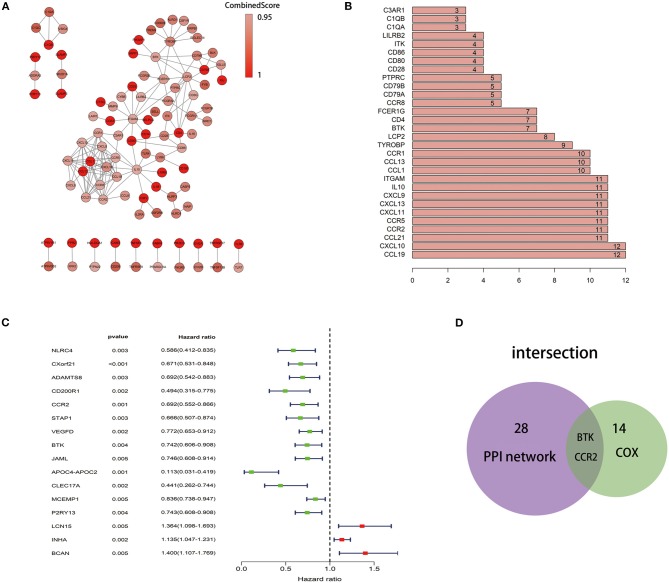
Protein–protein interaction network and univariate COX. **(A)** Interaction network constructed with the nodes with interaction confidence value >0.95. **(B)** The top 30 genes ordered by the number of nodes. **(C)** Univariate COX regression analysis with 379 DEGs, listing the top significant factors with *p* < 0.005. **(D)** Venn plot showing the common factors shared by leading 30 nodes in PPI and top significant factors in univariate COX.

To further explore the underlying mechanism, we constructed PPI network based on the STRING database using Cytoscape software [National Institute of General Medical Sciences (NIGMS) USA]. The interactions between 379 genes are shown in Figure 5A, and the bar plots were represented for the top 30 genes ranked by the number of nodes (Figure 5B). Univariate COX regression analysis for the survival of LUAD patients was performed to determine the significant factors among 379 DEGs (Figure 5C). And then, the intersection analysis between the leading nodes in PPI network and the top 16 factors ranked by the *p*-value of univariate COX regression was carried out, and only two factors, CCR2 and BTK, were overlapping from the above analyses (Figure 5D).

### The Correlation of BTK Expression With the Survival and Classification of TNM Stages in LUAD Patients

**Figure 6:**
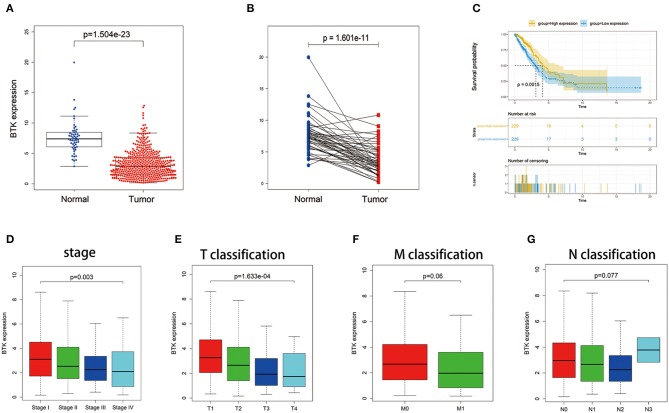
The differentiated expression of BTK in samples and correlation with survival and clinicopathological staging characteristics of LUAD patients. **(A)** Differentiated expression of BTK in the normal and tumor sample. Analyses were performed across all normal and tumor samples with *p* value closing to zero by Wilcoxon rank sum test. **(B)** Paired differentiation analysis for expression of BTK in the normal and tumor sample deriving from the same one patient (*p* = 1.601e−11 by the Wilcoxon rank sum test). **(C)** Survival analysis for LUAD patients with different BTK expression. Patients were labeled with high expression or low expression depending on the comparison with the median expression level. *p* = 0.0015 by log-rank test. **(D–G)** The correlation of BTK expression with clinicopathological staging characteristics. Wilcoxon rank sum or Kruskal–Wallis rank sum test served as the statistical significance test.

BTK played a key role in the intracellular signaling of B lymphocytes. Ibrutinib, a BTK inhibitor, was effective for the treatment of patients with lymphocytic malignancies. Instead, there were no delightful results obtained from the treatment of patients with solid tumors, for example, NSCLC and breast cancer. In the presented study, all LUAD samples were grouped into BTK high-expression group and BTK low-expression group compared with the BTK median expression. The survival analysis showed that LUAD patients with BTK high expression had longer survival than that of BTK low expression (Figure 6C). After that, the analysis of BTK combined with clinical characteristics was performed (Supplement Table 1), and Wilcoxon rank sum test revealed that the expression of BTK in the tumor samples was significantly lower than that in the normal samples (Figure 6A). Similar results were observed in the pairing analysis between the normal and tumor tissues derived from the same patient (Figure 6B). The above results clearly indicated that the expression of BTK in TME was positive correlation with the prognosis of LUAD patients. In particular, the expressions of BTK were declined along with the progression of TNM stages (Figures 6D–G).

### BTK Had Potential to Be an Indicator of TME Modulation

**Figure 7:**
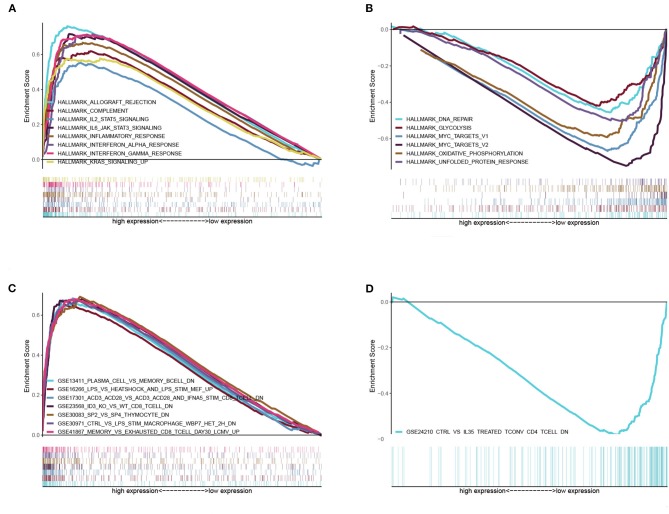
GSEA for samples with high BTK expression and low expression. **(A)** The enriched gene sets in HALLMARK collection by the high BTK expression sample. Each line representing one particular gene set with unique color, and up-regulated genes located in the left approaching the origin of the coordinates, by contrast the down-regulated lay on the right of x-axis. Only gene sets with NOM *p* < 0.05 and FDR *q* < 0.06 were considered significant. And only several leading gene sets were displayed in the plot. **(B)** The enriched gene sets in HALLMARK by samples with low BTK expression. **(C)** Enriched gene sets in C7 collection, the immunologic gene sets, by samples of high BTK expression. Only several leading gene sets are shown in plot. **(D)** Enriched gene sets in C7 by the low BTK expression.

Given the levels of BTK were negatively correlated with the survival and TNM stages of LUAD patients, GSEA was implemented in the high-expression and the low-expression groups compared with the median level of BTK expression, respectively. As shown in Figure 7A and Supplement Table 2, the genes in BTK high-expression group were mainly enriched in immune-related activities, such as allograft rejection, complement, and interferon response. As to BTK low-expression group, the genes were enriched in metabolic pathways, including glycolysis, oxidative phosphorylation, and typical tumor pathways (Figure 7B and Supplement Table 2). For C7 collection defined by MSigDB, the immunologic gene sets, multiple immune functional gene sets were enriched in the high BTK expression group (Figure 7C and Supplement Table 2). However, few gene sets were enriched in the low BTK expression group (Figure 7D and Supplement Table 2). These results suggested that BTK might be a potential indicator for the status of TME.

### Correlation of BTK With the Proportion of TICs

**Figure 8:**
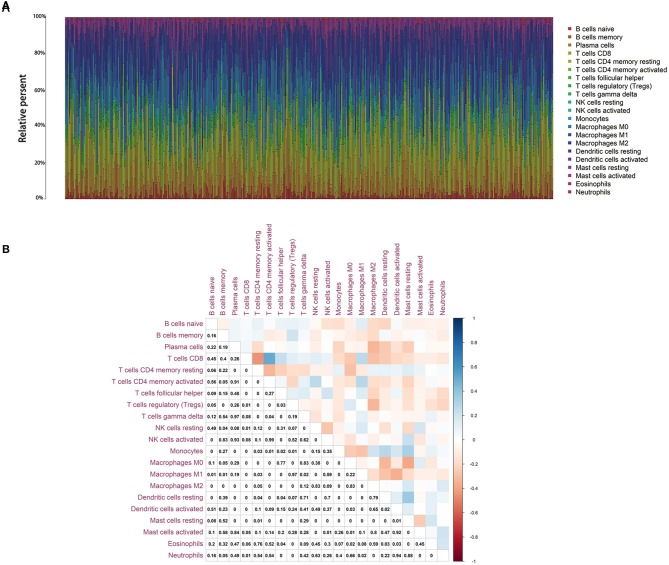
TIC profile in tumor samples and correlation analysis. **(A)** Barplot showing the proportion of 21 kinds of TICs in LUAD tumor samples. Column names of plot were sample ID. **(B)** Heatmap showing the correlation between 21 kinds of TICs and numeric in each tiny box indicating the *p* value of correlation between two kinds of cells. The shade of each tiny color box represented corresponding correlation value between two cells, and Pearson coefficient was used for significance test.

**Figure 9:**
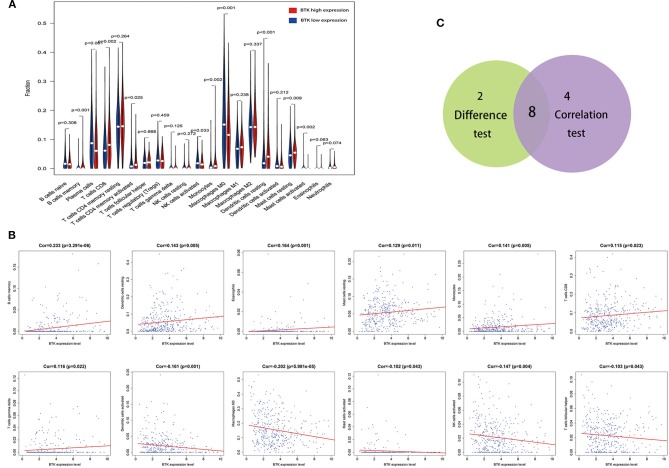
Correlation of TICs proportion with BTK expression. **(A)** Violin plot showed the ratio differentiation of 21 kinds of immune cells between LUAD tumor samples with low or high BTK expression relative to the median of BTK expression level, and Wilcoxon rank sum was used for the significance test. **(B)** Scatter plot showed the correlation of 12 kinds of TICs proportion with the BTK expression (*p* < 0.05). The red line in each plot was fitted linear model indicating the proportion tropism of the immune cell along with BTK expression, and Pearson coefficient was used for the correlation test. **(C)** Venn plot displayed eight kinds of TICs correlated with BTK expression codetermined by difference and correlation tests displayed in violin and scatter plots, respectively.

To further confirm the correlation of BTK expression with the immune microenvironment, the proportion of tumor-infiltrating immune subsets was analyzed using CIBERSORT algorithm, and 21 kinds of immune cell profiles in LUAD samples were constructed (Figure 8). The results from the difference and correlation analyses showed that a total of eight kinds of TICs were correlated with the expression of BTK (Figure 9, Supplement Figure 1, and Supplement Table 3). Among them, five kinds of TICs were positively correlated with BTK expression, including B-cell memory, CD8+ T cells, monocytes, resting dendritic cells, and resting mast cells; three kinds of TICs were negatively correlated with BTK expression, including activated NK cells, macrophage M0, and activated mast cells. These results further supported that the levels of BTK affected the immune activity of TME.

## Discussion

In the presented study, we attempted to identify TME-related genes that contributed to the survival and the classification of TNM stages in LUAD patients from the TCGA database. BTK was identified to be involved in immune activities. Importantly, a series of bioinformatics analysis indicated that BTK might be an indicator for the status of TME in LUAD patients.

TME played a critical role in the initiation and progression of tumorigenesis. It is of great benefit to explore the potential therapeutic targets contributing to remodeling of TME and fostering transition of TME from tumor-friendly to tumor-suppressed. A large number of studies had shed light on the importance of immune microenvironment in tumorigenesis. Our results from the transcriptome analysis upon LUAD data in TCGA database implied that the immune components in TME contributed to the prognosis of patients. Particularly, the proportion of immune and stromal components in TME significantly correlated with the progression of LUAD, such as invasion and metastasis. These results highlighted the significance of exploring the interaction between tumor cells and immune cells, which provided novel insight for developing much more effective treatment regimen. Recently, a great advancement has been made in immunotherapy, and immune checkpoint inhibitors (ICIs) have been approved as a first-line drug for patients with advanced NSCLC (17). However, programmed death 1 ligand, the most important factor, seemed not like a good indicator to determine whether NSCLC patients were suitable for immunotherapy (18). As a heterogeneous tumor, NSCLC was consistently known as a non-immunogenic tumor. Nevertheless, recent studies discovered the existence of tumor antigen-specific cytotoxicity and clonal TIL expansion of particular sites in NSCLC, which was opposed to the previous understanding (19). Besides, the abundance of TILs was significantly correlated with the 5-year survival rate of NSCLC, and the low counts of preoperative lymphocyte have been known as a poor prognostic signal for early-stage NSCLC patients (7, 8). Despite promising efficacy in NSCLC treatment demonstrated by ICIs, a wide variety of immune-related adverse events could not easily be ignored (20). Therefore, the universality of ICIs as well as the susceptibility to immune-related adverse events was a tough problem, and it was necessary to investigate some novel candidates for the immunotherapy of NSCLC. Here, we embarked from the transcriptomic analysis of LUAD in TCGA database, which revealed that the decreased expression of BTK was significantly associated with the advanced clinicopathological characteristics (clinical stages and distant metastasis) and poor prognosis. Accordingly, it suggested that BTK might be a potential prognostic marker and a therapeutic target for TME in LUAD.

BTK is a non-receptor tyrosine kinase and a member of Tec kinase family. As a key component of the upstream in BCR signaling, BTK played a vital role in the proliferation and differentiation of B cells (13, 14). A small-molecule inhibitor of BTK, ibrutinib, had been used for the patients with hematological malignancies (21, 22). Recently, ibrutinib was expanded to treatment of some solid tumors, including pancreatic cancer, breast cancer, and NSCLC. However, it received no comforting effects as those of hematological malignancies (15, 23, 24). Our results suggested that the expression of BTK was decreased in the advancing stages of LUAD patients, which seemed to be inconsistent with hematological malignancies. Similarly, some studies had reported that BTK might serve as a downstream effector in KRAS- and EGFR-activated signals in NSCLC, which might be explained by the existence of the distinct isoform of BTK in NSCLC (25, 26). Therefore, the discordance of response to BTK inhibitor implied that BTK seemed to play an antitumor role in LUAD. Further supports were derived from a series of studies, which indicated that BTK could modulate p53 activity to enhance tumor suppressor responses referring its antineoplastic properties (27–29). Thus, BTK might play a double-face role in tumor, either promoting survival or inducing apoptosis. Besides, it has been reported that BTK might be involved in regulating macrophage polarization in TME. Therefore, we further analyzed the relationship between BTK expression and TME. The GSEA results showed that immune-related signaling pathways, such as allograft rejection, complement, and interferon response, were significantly enriched in the BTK high-expression group. In the BTK low-expression group, metabolic pathways including glycolysis, oxidative phosphorylation, and typical tumor pathways were enriched. These results implied that BTK might participate in the status conversion of TME from immune-dominant to metabolic-dominant. Accumulated evidence had elucidated that BTK might be correlative to the metabolism. Ibrutinib promoted the uptake of glucose and glutamine and inhibited the synthesis of free fatty acid, which might be carried out via p53 signals (28, 30). To a certain degree, our data also showed that the balance between typical tumor pathways and vigorous glycolysis metabolism would affect the immunity status. The disorder of the balance could be reflected by the correlation of BTK low expression with metabolism. Further analysis of TIC supported this view. Accordingly, the downregulation of BTK along with the advancing stage of LUAD, the conversion of TME from immune-predominant to metabolic-dominant status, and the reduction of antitumor TICs supported that BTK might play an antitumor role in LUAD.

It was well-known that BTK was crucial for the functions of B lymphocytes. In the presented article, the CIBERSORT analysis for the proportion of TICs revealed that B-cell memory was positively correlated with BTK expression in LUAD patients. Regarding the role of B cells in cancer, there were some contradictory statements. A study indicated B cell as the promoter of carcinogenesis by inducing immunosuppression. However, the other study showed that CD40-activated B cell was a vigorous antigen-presenting cell and was able to induce the effect of antitumor immunity (31). A recent study revealed a correlation between the amounts of tumor-infiltrating B cells and the survival of LUAD patients with specific mutation driver, which suggested that tumor-infiltrating B cells might be a symbol for the specific mutation in lung cancer cells (32). Therefore, the positive correlation between the amounts of B-cell memory and BTK expression in LUAD patients suggested that BTK might be responsible for the preservation of immune-active status in TME.

Using ESTIMATE algorithm, we determined the TME-related genes in LUAD through the functional enrichment analysis of LUAD samples in TCGA database. BTK was a potential prognostic factor for LUAD patients. More interestingly, BTK might be an indicator for the conversion of TME status from immune-dominant to metabolic-dominant. Therefore, further investigation should be conducted to clarify the accuracy of a combined analysis of BTK expression, the amounts of tumor-infltrating B-cell isoforms, and the types of mutation-driven prior to BTK inhibitor treatment for LUAD patients.

## Materials and Methods

### Raw Data

Transcriptome RNA-seq data of 551 LUAD cases (normal samples, 54 cases; tumor samples, 497 cases) and the corresponding clinical data were downloaded from TCGA database (https://portal.gdc.cancer.gov/) with level 3.

### Generation of ImmuneScore, StromalScore, and ESTIMATEScore

ESTIMATE algorithm by feat of R language version 3.5.1 loaded with estimate package (33) was used to estimate the ratio of immune-stromal component in TME for each sample, exhibited in the form of three kinds of scores: ImmuneScore, StromalScore, and ESTIMATEScore, which positively correlated with the ratio of immune, stromal, and the sum of both, respectively, which means the higher the respective score, the larger the ratio of the corresponding component in TME.

### Survival Analysis

R language loaded with package survival and survminer was applied for the survival analysis. 458 tumor samples out of 497 cases had a detailed survival time record, with time span from 0 to 18.7 years, which were used for survival analysis. Kaplan–Meier method was used to plot the survival curve, and log rank as the statistical significance test; *p* < 0.05 was considered significant.

### Generation of DEGs Between High-Score and Low-Score Groups Regarding ImmuneScore and StromalScore

497 tumor samples were labeled with high score or low score depending on the comparison to the median score in regarding ImmuneScore and StromalScore, respectively. Package limma was used to perform differentiation analysis of the gene expression, and DEGs were generated by the comparison between the high-score samples vs. the low-score samples. DEGs with fold change larger than 1 after transformation of log_2_ (high-score group/low-score group) and false discovery rate (FDR) <0.05 were considered significant.

### GO and KEGG Enrichment Analysis

GO and KEGG enrichment analyses using 379 DEGs were performed with R language with the aid of packages clusterProfiler, enrichplot, and ggplot2. Only terms with both *p*- and *q*-value of <0.05 were considered significantly enriched.

### Heatmaps

Heatmaps of DEGs were produced by R language with package pheatmap.

### Difference Analysis of Scores With Clinical Stages

The clinicopathological characteristics data corresponding to the LUAD samples were downloaded from TCGA. The analysis was performed by R language, and Wilcoxon rank sum or Kruskal–Wallis rank sum test as the significance test depending on the number of clinical stages for comparison.

### PPI Network Construction

PPI network was constructed by STRING database, followed by reconstruction with Cytoscape of version 3.6.1. Nodes with confidence of interactive relationship larger than 0.95 were used for building network.

### COX Regression Analysis

R language loaded with package survival was used for univariate COX regression. The top 16 genes ordered by *p* value from small to large in univariate COX were shown in the plot.

### Gene Set Enrichment Analysis

Hallmark and C7 gene sets v6.2 collections were downloaded from Molecular Signatures Database as the target sets with which GSEA performed using the software gsea-3.0 downloaded from Broad Institute. The whole transcriptome of all tumor samples was used for GSEA, and only gene sets with NOM *p* < 0.05 and FDR *q* < 0.06 were considered as significant.

### TICs Profile

CIBERSORT computational method was applied for estimating the TIC abundance profile in all tumor samples, which followed by quality filtering that only 421 tumor samples with *p* < 0.05 were selected for the following analysis.

## Data Availability Statement

The datasets generated and analyzed during this study are available in the TCGA database (https://portal.gdc.cancer.gov).

## Author Contributions

K-WB, X-GW, and BL came up with the design and conception. The data analysis and visualization were conducted by K-WB, X-GW and X-XQ. The original writing of the draft and its editing were by K-WB and BL.

### Conflict of Interest

The authors declare that the research was conducted in the absence of any commercial or financial relationships that could be construed as a potential conflict of interest.

## Supplementary Material

Supplement Figure 1 TICs with non-significant correlation with the expression of BTK.

Supplement Table 1 Clinicopathological characteristics statistics of LUAD patients from TCGA.

Supplement Table 2 Enriched gene sets.

Supplement Table 3 TICs co-determined by difference test and correlation test.

## Abbreviations

TME: tumor microenvironment
LUAD: lung adenocarcinoma
PPI: protein–protein interaction
DEG: differentially expressed gene
BTK: Bruton tyrosine kinase
GSEA: Gene Set Enrichment Analysis
NSCLC: non–small cell lung cancer
TIC: tumor-infiltrating immune cell
TIL: tumor-infiltrating lymphocyte
ICI: immune checkpoint inhibitor.
