# Supplementary figures and images for: BTK Has Potential to Be a Prognostic Factor for Lung Adenocarcinoma and an Indicator for Tumor Microenvironment Remodeling: A Study Based on TCGA Data Mining
Source: Front Oncol. 2020 Apr 15;10:424. doi: 10.3389/fonc.2020.00424 (PMC7175916; doi:10.3389/fonc.2020.00424)

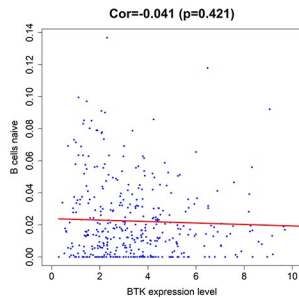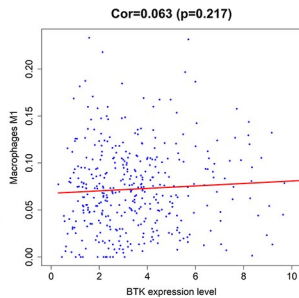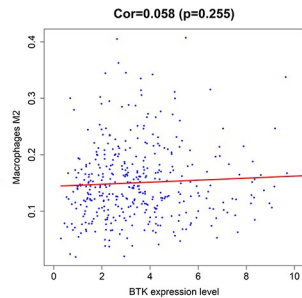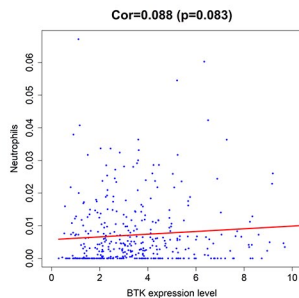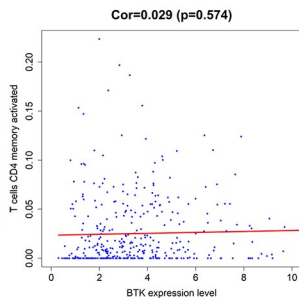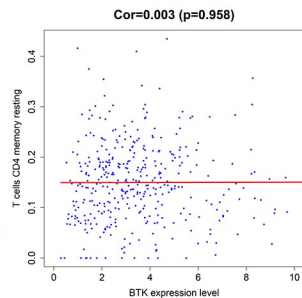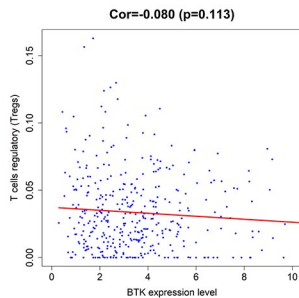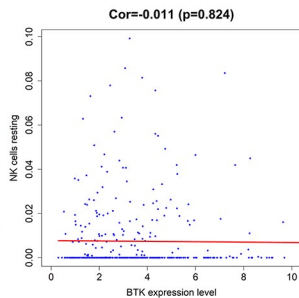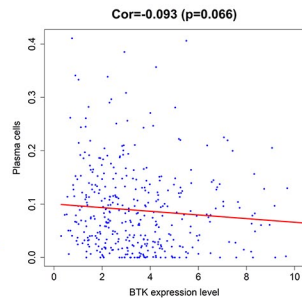

Supplement: Supplement Figure 1 — TICs with non-significant correlation with the expression of BTK. [file Data_Sheet_1.PDF]
