# Supplementary material for: BTK Has Potential to Be a Prognostic Factor for Lung Adenocarcinoma and an Indicator for Tumor Microenvironment Remodeling: A Study Based on TCGA Data Mining
Source: Front Oncol. 2020 Apr 15;10:424. doi: 10.3389/fonc.2020.00424 (PMC7175916; doi:10.3389/fonc.2020.00424)
Supplement: Supplement Table 1 — Clinicopathological characteristics statistics of LUAD patients from TCGA. [file Table_1.DOCX]

Supplement Table 1. Clinicopathological characteristics statistics of LUAD patients from TCGA.

| Clinical characteristics |  | | Total  (486) | | % |
| --- | --- | --- | --- | --- | --- |
| Age at diagnosis (y)  Gender  Stage  T classification  M classification  N classification | young age (<=60)  old age (>60)  Male  Female  Ⅰ  Ⅱ  Ⅲ  Ⅳ  T1  T2  T3  T4  M0  M1  N0  N1  N2  N3 | 155  312  222  264  262  112  79  25  163  260  41  19  333  24  312  90  70  2 | | 33.2  66.8  45.7  54.3  54.8  23.4  16.5  5.2  33.7  53.8  8.5  3.9  93.3  6.7  65.8  19  14.8  0.4 | |
