# Supplementary material for: BTK Has Potential to Be a Prognostic Factor for Lung Adenocarcinoma and an Indicator for Tumor Microenvironment Remodeling: A Study Based on TCGA Data Mining
Source: Front Oncol. 2020 Apr 15;10:424. doi: 10.3389/fonc.2020.00424 (PMC7175916; doi:10.3389/fonc.2020.00424)
Supplement: Supplement Table 2 — Enriched gene sets. [file Table_2.DOCX]

Supplement Table 2. Enriched gene sets.

| MSigDB collection | Gene set name | NES | NOM p-val | FDR q-val |
| --- | --- | --- | --- | --- |
| h.all.v6.2.symbols.gmt  BTK high expression    BTK low expression  c7.all.v6.2.symbols.gmt  BTK high expression  BTK low expression | HALLMARK_ALLOGRAFT_REJECTION  HALLMARK_COMPLEMENT  HALLMARK_KRAS_SIGNALING_UP  HALLMARK_IL2_STAT5_SIGNALING  HALLMARK_IL6_JAK_STAT3_SIGNALING  HALLMARK_INFLAMMATORY_RESPONSE  HALLMARK_INTERFERON_GAMMA_RESPONSE  HALLMARK_INTERFERON_ALPHA_RESPONSE  HALLMARK_MYC_TARGETS_V2  HALLMARK_MYC_TARGETS_V1  HALLMARK_UNFOLDED_PROTEIN_RESPONSE  HALLMARK_OXIDATIVE_PHOSPHORYLATION  HALLMARK_GLYCOLYSIS  HALLMARK_DNA_REPAIR  GSE23568_ID3_KO_VS_WT_CD8_TCELL_DN  GSE17301_ACD3_ACD28_VS_ACD3_ACD28_AND_IFNA5_STIM_CD8_TCELL_DN  GSE16266_LPS_VS_HEATSHOCK_AND_LPS_STIM_MEF_UP  GSE41867_MEMORY_VS_EXHAUSTED_CD8_TCELL_DAY30_LCMV_UP  GSE30083_SP2_VS_SP4_THYMOCYTE_DN  GSE30971_CTRL_VS_LPS_STIM_MACROPHAGE_WBP7_HET_2H_DN  GSE13411_PLASMA_CELL_VS_MEMORY_BCELL_DN  GSE24210_CTRL_VS_IL35_TREATED_TCONV_CD4_TCELL_DN | 2.469  2.408  2.375  2.366  2.365  2.356  2.190  1.961  -2.039  -2.001  -1.884  -1.803  -1.738  -1.719  2.781  2.732  2.718  2.712  2.709  2.688  2.686  -2.107 | 0  0  0  0  0  0  0.002  0.006  0  0  0.004  0.021  0.008  0.023  0  0  0  0  0  0  0  0 | 0  0  0  0  0  0  0.001  0.009  0.016  0.013  0.027  0.040  0.056  0.053  0  0  0  0  0  0  0  0.035 |

NES: normalized enrichment score; NOM: nominal p-value; FDR: false discovery rate. Gene sets with NOM p-value less than 0.05 and FDR q-value less than 0.06 were considered as statistical significance. Only several leading sets enriched in BTK high expression both in HALLMARK and C7 were listed here due to the large number of enriched gene sets.
