# Supplementary material for: BTK Has Potential to Be a Prognostic Factor for Lung Adenocarcinoma and an Indicator for Tumor Microenvironment Remodeling: A Study Based on TCGA Data Mining
Source: Front Oncol. 2020 Apr 15;10:424. doi: 10.3389/fonc.2020.00424 (PMC7175916; doi:10.3389/fonc.2020.00424)
Supplement: Supplement Table 3 — TICs co-determined by difference test and correlation test. [file Table_3.docx]

Supplement Table 3. TICs co-determined by difference test and correlation test

| TICs | Correlation test（p-value） | | Difference test（p-value） |
| --- | --- | --- | --- |
| B cells memory  T cells CD8  NK cells activated  Monocytes  Macrophage M0  Dendritic cells resting  Mast cells resting  Mast cells activated | 0.233 (<0.001)  0.115 ( 0.023)  -0.147 (0.004)  0.141 (0.005)  -0.202 (<0.001)  0.143 (0.005)  0.129 (0.011)  -0.102 (0.043) | <0.001  0.002  0.033  0.002  0.001  <0.001  0.009  0.002 | |
